# *catena*-Poly[[(5-phenyl-2,2′-bipyridine-κ^2^ *N*,*N*′)copper(I)]-μ-thio­cyanido-κ^2^ *N*:*S*]

**DOI:** 10.1107/S1600536812000682

**Published:** 2012-01-18

**Authors:** Shuxin Cui, Minghui Zuo, Jingping Zhang, Yulong Zhao, Hui Wang

**Affiliations:** a College of Chemistry and Chemical Engineering, Mu Danjiang Normal University, Mu Danjiang 157012, People’s Republic of China; b Faculty of Chemistry, Northeast Normal University, Changchun 130024, People’s Republic of China

## Abstract

The title compound, [Cu(NCS)(C_16_H_12_N_2_)]_*n*_, was synthesised under hydro­thermal conditions. The Cu^I^ ion shows distorted tetra­hedral geometry being coordinated by two N atoms from a 5-phenyl-2,2′-bipyridine ligand and by the N and S atoms from two different thio­cyanate anions. The Cu^I^ ions are bridged by thio­cyanide groups, forming a one-dimensional coordination polymer along the *b* axis. The crystal packing is through van der Waals contacts and C—H⋯π inter­actions.

## Related literature

For applications of coordination metal complexes, see: Kong *et al.* (2008 ▶); Ohba *et al.* (2008 ▶). For related compounds, see Chen *et al.* (2009 ▶); Cui *et al.* (2011 ▶); Zhang *et al.* (2008 ▶).
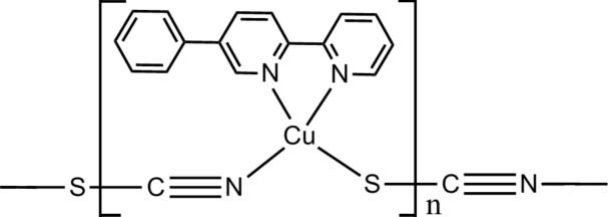


## Experimental

### 

#### Crystal data

[Cu(NCS)(C_16_H_12_N_2_)]
*M* *_r_* = 353.90
Orthorhombic, 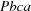

*a* = 7.7978 (9) Å
*b* = 10.7744 (12) Å
*c* = 35.325 (4) Å
*V* = 2967.8 (6) Å^3^
*Z* = 8
Mo *K*α radiation
μ = 1.61 mm^−1^
*T* = 153 K
0.42 × 0.09 × 0.06 mm

#### Data collection

Siemens SMART CCD area-detector diffractometer
Absorption correction: multi-scan (*SADABS*; Sheldrick, 2004 ▶) *T*_min_ = 0.840, *T*_max_ = 0.914
15488 measured reflections
2932 independent reflections
2212 reflections with *I* > 2σ(*I*)
*R*_int_ = 0.033

#### Refinement

*R*[*F* ^2^ > 2σ(*F* ^2^)] = 0.035
*wR*(*F* ^2^) = 0.091
*S* = 1.03
2932 reflections
199 parameters
H-atom parameters constrained
Δρ_max_ = 0.32 e Å^−3^
Δρ_min_ = −0.20 e Å^−3^

### 

Data collection: *SMART* (Bruker, 2007 ▶); cell refinement: *SAINT* (Bruker, 2007 ▶); data reduction: *SAINT*; program(s) used to solve structure: *SHELXS97* (Sheldrick, 2008 ▶); program(s) used to refine structure: *SHELXL97* (Sheldrick, 2008 ▶); molecular graphics: *XP* (Sheldrick, 2008 ▶); software used to prepare material for publication: *SHELXL97*.

## Supplementary Material

Crystal structure: contains datablock(s) I, global. DOI: 10.1107/S1600536812000682/kp2379sup1.cif

Structure factors: contains datablock(s) I. DOI: 10.1107/S1600536812000682/kp2379Isup2.hkl

Additional supplementary materials:  crystallographic information; 3D view; checkCIF report

## Figures and Tables

**Table 1:** Selected bond lengths (Å)

Cu1—N1	1.917 (2)
Cu1—N2	2.079 (2)
Cu1—N3	2.121 (2)
Cu1—S1	2.3313 (9)

**Table 2:** Hydrogen-bond geometry (Å, °) *Cg* is the centroid of the C12–C17 ring.

*D*—H⋯*A*	*D*—H	H⋯*A*	*D*⋯*A*	*D*—H⋯*A*
C17—H17⋯*Cg*^i^	0.93	2.92	3.757 (3)	150

Symmetry code: (i) 
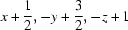
.

**Fig. 1.**
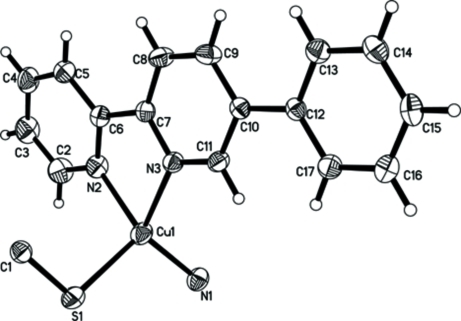
Complex (I), with the atom-numbering scheme. Displacement ellipsoids are drawn at the 50% probability level and H atoms are shown as small of arbitrary radii.

**Fig. 2.**
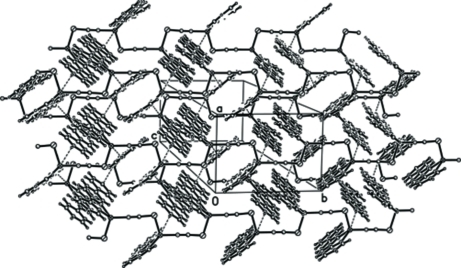
A perspective view of polymer chain of complex (I).

## supplementary crystallographic information

### Comment

The title compound [Cu(SCN)(5-ph-2,2'-bpy)]_n_(I), was synthesised under hydrothermal conditions. In the complex, the Cu^I^ ion shows distorted tetrahedral geometry coordinated by two N atoms from a 5-ph-2,2,-bpy ligand, N and S atoms from the different thiocyanate anions (Fig.1 and Table 1). The Cu^I^ions are bridged by thiocyanide groups to form a one-dimensional coordination polymeric along the *b* axis (Fig. 2). Crystal packing is through van der Waals contacts and C-H···π interaction [C17-H···Cg(C12→C17)symmetry operation: 1/2+x,3/2-y,1-z with geometric parameters H···Cg of 2.92 Å, C17···Cg of 3.757 (3) Å, and C17-H···Cg of 150 °].

### Experimental

A mixture of Cu(Ac)_2_ (0.086 g, 0.64 mmol), 5-ph-2,2'-bpy (0.0231 g, 0.1 mmol), KSCN (0.059 g, 0.6 mmol), and water (8 mL) was added to a 15-mL Teflon-lined autoclave and heated at 443 K for 3 d. The autoclave was then cooled to room temperature. Red block crystals of (I) deposited on the wall of container were collected and air-dried.

### Refinement

Hydrogen atoms bound to carbon were placed in calculated positions and refined using a riding model with an isotropic displacement parameter fixed at 1.2 times *U*_eq_ for the atom to which they are attached.

### Crystal data

**Table:**

[Cu(NCS)(C_16_H_12_N_2_)]	*F*(000) = 1440
*M**_r_* = 353.90	*D*_x_ = 1.584 Mg m^−^^3^
Orthorhombic, *P**b**c**a*	Mo *K*α radiation, λ = 0.71073 Å
Hall symbol: -P 2ac 2ab	Cell parameters from 15488 reflections
*a* = 7.7978 (9) Å	θ = 2.3–26.0°
*b* = 10.7744 (12) Å	µ = 1.61 mm^−^^1^
*c* = 35.325 (4) Å	*T* = 153 K
*V* = 2967.8 (6) Å^3^	Block, red
*Z* = 8	0.42 × 0.09 × 0.06 mm

### Data collection

**Table:**

Siemens SMART CCD area-detector diffractometer	2932 independent reflections
Radiation source: fine-focus sealed tube	2212 reflections with *I* > 2σ(*I*)
graphite	*R*_int_ = 0.033
Detector resolution: 9 pixels mm^-1^	θ_max_ = 26.0°, θ_min_ = 2.3°
ω scans	*h* = −9→9
Absorption correction: multi-scan (*SADABS*; Sheldrick, 2004)	*k* = −13→10
*T*_min_ = 0.840, *T*_max_ = 0.914	*l* = −43→35
15488 measured reflections	

### Refinement

**Table:**

Refinement on *F*^2^	Primary atom site location: structure-invariant direct methods
Least-squares matrix: full	Secondary atom site location: difference Fourier map
*R*[*F*^2^ > 2σ(*F*^2^)] = 0.035	Hydrogen site location: inferred from neighbouring sites
*wR*(*F*^2^) = 0.091	H-atom parameters constrained
*S* = 1.03	*w* = 1/[σ^2^(*F*_o_^2^) + (0.0444*P*)^2^ + 0.9913*P*] where *P* = (*F*_o_^2^ + 2*F*_c_^2^)/3
2932 reflections	(Δ/σ)_max_ = 0.001
199 parameters	Δρ_max_ = 0.32 e Å^−^^3^
0 restraints	Δρ_min_ = −0.20 e Å^−^^3^

### Special details

**Table:**

Geometry. All e.s.d.'s (except the e.s.d. in the dihedral angle between two l.s. planes) are estimated using the full covariance matrix. The cell e.s.d.'s are taken into account individually in the estimation of e.s.d.'s in distances, angles and torsion angles; correlations between e.s.d.'s in cell parameters are only used when they are defined by crystal symmetry. An approximate (isotropic) treatment of cell e.s.d.'s is used for estimating e.s.d.'s involving l.s. planes.
Refinement. Refinement of *F*^2^ against ALL reflections. The weighted *R*-factor *wR* and goodness of fit *S* are based on *F*^2^, conventional *R*-factors *R* are based on *F*, with *F* set to zero for negative *F*^2^. The threshold expression of *F*^2^ > σ(*F*^2^) is used only for calculating *R*-factors(gt) *etc*. and is not relevant to the choice of reflections for refinement. *R*-factors based on *F*^2^ are statistically about twice as large as those based on *F*, and *R*- factors based on ALL data will be even larger.

### Fractional atomic coordinates and isotropic or equivalent isotropic displacement parameters (Å^2^)

**Table:**

	*x*	*y*	*z*	*U*_iso_*/*U*_eq_	
Cu1	1.11691 (4)	0.67597 (3)	0.345104 (9)	0.04739 (14)	
S1	1.40271 (9)	0.61263 (7)	0.34368 (3)	0.0578 (2)	
N1	1.1156 (3)	0.8538 (2)	0.34377 (6)	0.0470 (6)	
C1	1.3879 (3)	0.4601 (3)	0.34412 (7)	0.0402 (6)	
N3	0.9620 (3)	0.59247 (18)	0.38739 (6)	0.0373 (5)	
C12	0.8393 (3)	0.6026 (2)	0.49059 (6)	0.0325 (5)	
C7	0.8656 (3)	0.4982 (2)	0.37430 (7)	0.0351 (5)	
C10	0.8445 (3)	0.5642 (2)	0.45016 (6)	0.0327 (5)	
N2	0.9760 (3)	0.5504 (2)	0.31300 (6)	0.0460 (5)	
C6	0.8833 (3)	0.4689 (2)	0.33359 (7)	0.0378 (6)	
C8	0.7568 (3)	0.4343 (3)	0.39843 (7)	0.0457 (6)	
H8	0.6907	0.3691	0.3893	0.055*	
C15	0.8303 (4)	0.6706 (3)	0.56739 (7)	0.0461 (7)	
H15	0.8272	0.6938	0.5927	0.055*	
C11	0.9497 (3)	0.6232 (2)	0.42376 (7)	0.0381 (6)	
H11	1.0163	0.6891	0.4323	0.046*	
C17	0.9373 (3)	0.7005 (2)	0.50432 (7)	0.0423 (6)	
H17	1.0077	0.7444	0.4878	0.051*	
C16	0.9318 (3)	0.7338 (3)	0.54203 (8)	0.0471 (7)	
H16	0.9979	0.8002	0.5504	0.057*	
C14	0.7336 (3)	0.5726 (3)	0.55461 (7)	0.0465 (6)	
H14	0.6649	0.5287	0.5714	0.056*	
C9	0.7469 (3)	0.4675 (2)	0.43580 (7)	0.0451 (6)	
H9	0.6733	0.4244	0.4518	0.054*	
C5	0.8093 (4)	0.3650 (3)	0.31698 (8)	0.0490 (7)	
H5	0.7476	0.3088	0.3316	0.059*	
C13	0.7381 (3)	0.5388 (2)	0.51663 (7)	0.0426 (6)	
H13	0.6720	0.4722	0.5084	0.051*	
C3	0.9202 (4)	0.4297 (3)	0.25767 (9)	0.0643 (9)	
H3	0.9332	0.4194	0.2317	0.077*	
C4	0.8281 (4)	0.3458 (3)	0.27870 (9)	0.0621 (9)	
H4	0.7789	0.2768	0.2672	0.074*	
C2	0.9924 (4)	0.5287 (3)	0.27587 (8)	0.0607 (8)	
H2	1.0568	0.5844	0.2616	0.073*	

### Atomic displacement parameters (Å^2^)

**Table:**

	*U*^11^	*U*^22^	*U*^33^	*U*^12^	*U*^13^	*U*^23^
Cu1	0.0601 (2)	0.0344 (2)	0.0477 (2)	−0.00445 (15)	0.00674 (16)	0.00251 (14)
S1	0.0532 (4)	0.0325 (4)	0.0878 (6)	−0.0041 (3)	0.0084 (4)	−0.0025 (4)
N1	0.0582 (15)	0.0369 (13)	0.0459 (14)	0.0008 (11)	−0.0004 (11)	0.0028 (10)
C1	0.0414 (14)	0.0413 (16)	0.0378 (14)	−0.0003 (11)	0.0025 (11)	−0.0002 (11)
N3	0.0432 (11)	0.0341 (11)	0.0346 (11)	−0.0032 (9)	0.0032 (9)	0.0012 (9)
C12	0.0312 (12)	0.0312 (12)	0.0349 (13)	0.0072 (10)	0.0011 (10)	0.0035 (10)
C7	0.0361 (12)	0.0336 (13)	0.0357 (13)	0.0044 (11)	−0.0020 (10)	0.0018 (10)
C10	0.0323 (12)	0.0324 (12)	0.0334 (13)	0.0048 (10)	−0.0004 (10)	0.0035 (10)
N2	0.0554 (13)	0.0483 (13)	0.0344 (12)	−0.0005 (11)	−0.0003 (10)	0.0018 (10)
C6	0.0387 (13)	0.0382 (14)	0.0365 (13)	0.0040 (11)	−0.0043 (11)	−0.0002 (11)
C8	0.0485 (15)	0.0470 (15)	0.0415 (14)	−0.0155 (13)	−0.0023 (12)	−0.0002 (12)
C15	0.0520 (15)	0.0526 (17)	0.0337 (14)	0.0146 (14)	0.0011 (12)	−0.0028 (12)
C11	0.0446 (14)	0.0335 (13)	0.0364 (14)	−0.0058 (11)	0.0010 (11)	−0.0011 (11)
C17	0.0482 (14)	0.0396 (15)	0.0392 (14)	−0.0019 (12)	0.0032 (11)	0.0023 (12)
C16	0.0535 (16)	0.0406 (15)	0.0473 (16)	0.0018 (13)	−0.0021 (12)	−0.0062 (12)
C14	0.0467 (15)	0.0547 (17)	0.0381 (14)	0.0032 (14)	0.0070 (12)	0.0049 (13)
C9	0.0443 (14)	0.0525 (16)	0.0386 (14)	−0.0143 (13)	0.0010 (12)	0.0051 (12)
C5	0.0486 (15)	0.0484 (16)	0.0501 (17)	−0.0032 (13)	−0.0006 (13)	−0.0068 (13)
C13	0.0436 (14)	0.0425 (15)	0.0416 (14)	−0.0008 (12)	0.0026 (12)	0.0012 (11)
C3	0.076 (2)	0.080 (2)	0.0370 (16)	0.0006 (19)	−0.0013 (15)	−0.0126 (16)
C4	0.0609 (18)	0.066 (2)	0.059 (2)	0.0003 (16)	−0.0046 (16)	−0.0277 (16)
C2	0.074 (2)	0.071 (2)	0.0367 (16)	−0.0054 (17)	0.0034 (14)	0.0021 (14)

### Geometric parameters (Å, °)

**Table:**

Cu1—N1	1.917 (2)	C8—H8	0.9300
Cu1—N2	2.079 (2)	C15—C14	1.374 (4)
Cu1—N3	2.121 (2)	C15—C16	1.376 (4)
Cu1—S1	2.3313 (9)	C15—H15	0.9300
S1—C1	1.648 (3)	C11—H11	0.9300
N1—C1^i^	1.145 (4)	C17—C16	1.380 (4)
C1—N1^ii^	1.145 (4)	C17—H17	0.9300
N3—C11	1.330 (3)	C16—H16	0.9300
N3—C7	1.346 (3)	C14—C13	1.390 (3)
C12—C17	1.390 (3)	C14—H14	0.9300
C12—C13	1.393 (3)	C9—H9	0.9300
C12—C10	1.487 (3)	C5—C4	1.376 (4)
C7—C8	1.385 (3)	C5—H5	0.9300
C7—C6	1.479 (3)	C13—H13	0.9300
C10—C9	1.386 (3)	C3—C2	1.367 (4)
C10—C11	1.395 (3)	C3—C4	1.373 (5)
N2—C2	1.339 (3)	C3—H3	0.9300
N2—C6	1.350 (3)	C4—H4	0.9300
C6—C5	1.390 (4)	C2—H2	0.9300
C8—C9	1.370 (4)		
			
N1—Cu1—N2	129.35 (9)	C16—C15—H15	120.6
N1—Cu1—N3	115.99 (9)	N3—C11—C10	125.1 (2)
N2—Cu1—N3	78.88 (8)	N3—C11—H11	117.5
N1—Cu1—S1	107.29 (7)	C10—C11—H11	117.5
N2—Cu1—S1	107.64 (7)	C16—C17—C12	121.1 (2)
N3—Cu1—S1	115.82 (6)	C16—C17—H17	119.4
C1—S1—Cu1	102.99 (9)	C12—C17—H17	119.4
C1^i^—N1—Cu1	177.7 (2)	C15—C16—C17	121.2 (3)
N1^ii^—C1—S1	177.1 (2)	C15—C16—H16	119.4
C11—N3—C7	118.7 (2)	C17—C16—H16	119.4
C11—N3—Cu1	127.99 (17)	C15—C14—C13	120.3 (2)
C7—N3—Cu1	113.34 (15)	C15—C14—H14	119.8
C17—C12—C13	117.1 (2)	C13—C14—H14	119.8
C17—C12—C10	122.1 (2)	C8—C9—C10	121.2 (2)
C13—C12—C10	120.8 (2)	C8—C9—H9	119.4
N3—C7—C8	120.3 (2)	C10—C9—H9	119.4
N3—C7—C6	116.3 (2)	C4—C5—C6	119.4 (3)
C8—C7—C6	123.3 (2)	C4—C5—H5	120.3
C9—C10—C11	114.9 (2)	C6—C5—H5	120.3
C9—C10—C12	123.0 (2)	C14—C13—C12	121.5 (2)
C11—C10—C12	122.1 (2)	C14—C13—H13	119.3
C2—N2—C6	117.8 (2)	C12—C13—H13	119.3
C2—N2—Cu1	126.7 (2)	C2—C3—C4	118.3 (3)
C6—N2—Cu1	114.36 (17)	C2—C3—H3	120.8
N2—C6—C5	121.3 (2)	C4—C3—H3	120.8
N2—C6—C7	115.8 (2)	C3—C4—C5	119.3 (3)
C5—C6—C7	122.9 (2)	C3—C4—H4	120.4
C9—C8—C7	119.9 (2)	C5—C4—H4	120.4
C9—C8—H8	120.1	N2—C2—C3	123.9 (3)
C7—C8—H8	120.1	N2—C2—H2	118.1
C14—C15—C16	118.8 (2)	C3—C2—H2	118.1
C14—C15—H15	120.6		
			
N1—Cu1—S1—C1	−178.99 (11)	C8—C7—C6—N2	171.1 (2)
N2—Cu1—S1—C1	−36.25 (11)	N3—C7—C6—C5	171.7 (2)
N3—Cu1—S1—C1	49.68 (11)	C8—C7—C6—C5	−8.5 (4)
N1—Cu1—N3—C11	−45.7 (2)	N3—C7—C8—C9	0.2 (4)
N2—Cu1—N3—C11	−174.2 (2)	C6—C7—C8—C9	−179.6 (2)
S1—Cu1—N3—C11	81.5 (2)	C7—N3—C11—C10	0.6 (4)
N1—Cu1—N3—C7	133.67 (16)	Cu1—N3—C11—C10	179.92 (18)
N2—Cu1—N3—C7	5.13 (16)	C9—C10—C11—N3	−0.7 (4)
S1—Cu1—N3—C7	−99.22 (16)	C12—C10—C11—N3	179.1 (2)
C11—N3—C7—C8	−0.3 (3)	C13—C12—C17—C16	−1.0 (4)
Cu1—N3—C7—C8	−179.74 (18)	C10—C12—C17—C16	−179.3 (2)
C11—N3—C7—C6	179.4 (2)	C14—C15—C16—C17	0.2 (4)
Cu1—N3—C7—C6	0.0 (3)	C12—C17—C16—C15	0.5 (4)
C17—C12—C10—C9	−179.4 (2)	C16—C15—C14—C13	−0.4 (4)
C13—C12—C10—C9	2.2 (3)	C7—C8—C9—C10	−0.2 (4)
C17—C12—C10—C11	0.9 (3)	C11—C10—C9—C8	0.4 (4)
C13—C12—C10—C11	−177.5 (2)	C12—C10—C9—C8	−179.3 (2)
N1—Cu1—N2—C2	68.4 (3)	N2—C6—C5—C4	−1.2 (4)
N3—Cu1—N2—C2	−176.9 (3)	C7—C6—C5—C4	178.4 (3)
S1—Cu1—N2—C2	−63.2 (2)	C15—C14—C13—C12	−0.1 (4)
N1—Cu1—N2—C6	−124.53 (18)	C17—C12—C13—C14	0.7 (4)
N3—Cu1—N2—C6	−9.93 (18)	C10—C12—C13—C14	179.2 (2)
S1—Cu1—N2—C6	103.86 (17)	C2—C3—C4—C5	1.0 (5)
C2—N2—C6—C5	0.9 (4)	C6—C5—C4—C3	0.2 (5)
Cu1—N2—C6—C5	−167.4 (2)	C6—N2—C2—C3	0.4 (5)
C2—N2—C6—C7	−178.8 (2)	Cu1—N2—C2—C3	167.1 (2)
Cu1—N2—C6—C7	13.0 (3)	C4—C3—C2—N2	−1.4 (5)
N3—C7—C6—N2	−8.7 (3)		

Symmetry codes: (i) −*x*+5/2, *y*+1/2, *z*; (ii) −*x*+5/2, *y*−1/2, *z*.

### Hydrogen-bond geometry (Å, °)

**Table:**

Cg is the centroid of the C12–C17 ring.

**Table:**

*D*—H···*A*	*D*—H	H···*A*	*D*···*A*	*D*—H···*A*
C17—H17···Cg^iii^	0.93	2.92	3.757 (3)	150

Symmetry codes: (iii) *x*+1/2, −*y*+3/2, −*z*+1.
